# Central Neurocircuits Regulating Food Intake in Response to Gut Inputs—Preclinical Evidence

**DOI:** 10.3390/nu13030908

**Published:** 2021-03-11

**Authors:** Kirsteen N. Browning, Kaitlin E. Carson

**Affiliations:** Department of Neural and Behavioral Sciences, Penn State College of Medicine, Hershey, PA 17033, USA; kcarson2@pennstatehealth.psu.edu

**Keywords:** brainstem, vagus, feeding, gastrointestinal

## Abstract

The regulation of energy balance requires the complex integration of homeostatic and hedonic pathways, but sensory inputs from the gastrointestinal (GI) tract are increasingly recognized as playing critical roles. The stomach and small intestine relay sensory information to the central nervous system (CNS) via the sensory afferent vagus nerve. This vast volume of complex sensory information is received by neurons of the nucleus of the tractus solitarius (NTS) and is integrated with responses to circulating factors as well as descending inputs from the brainstem, midbrain, and forebrain nuclei involved in autonomic regulation. The integrated signal is relayed to the adjacent dorsal motor nucleus of the vagus (DMV), which supplies the motor output response via the efferent vagus nerve to regulate and modulate gastric motility, tone, secretion, and emptying, as well as intestinal motility and transit; the precise coordination of these responses is essential for the control of meal size, meal termination, and nutrient absorption. The interconnectivity of the NTS implies that many other CNS areas are capable of modulating vagal efferent output, emphasized by the many CNS disorders associated with dysregulated GI functions including feeding. This review will summarize the role of major CNS centers to gut-related inputs in the regulation of gastric function with specific reference to the regulation of food intake.

## 1. Introduction

Sensory information from the gastrointestinal (GI) tract is relayed centrally via neural and humoral pathways; the central nervous system (CNS) integrates this large volume of sensory afferent information and coordinates a precise series of efferent responses to regulate food and caloric intake including the modulation of gastric motility, tone, and emptying, as well as intestinal motility, transit, secretion and absorption. This efferent output is regulated meticulously in order to maintain homeostasis based on current energy needs and visceral sensory stimuli. The advent of novel and emerging molecular and genetic labeling techniques implies that, as a field, we are in a better position to be able to potentially delineate the exact phenotype of neurons involved in distinct aspects of nutrient signaling, and to interrogate and manipulate those neurocircuits with spatial and temporal precision. Characterizing the central neurocircuits involved following activation of gut inputs, including their phenotypes, activation characteristics, and connectomes, will be important to understand the physiology and pathophysiology of food intake.

### 1.1. Afferent Inputs

**Figure 1:**
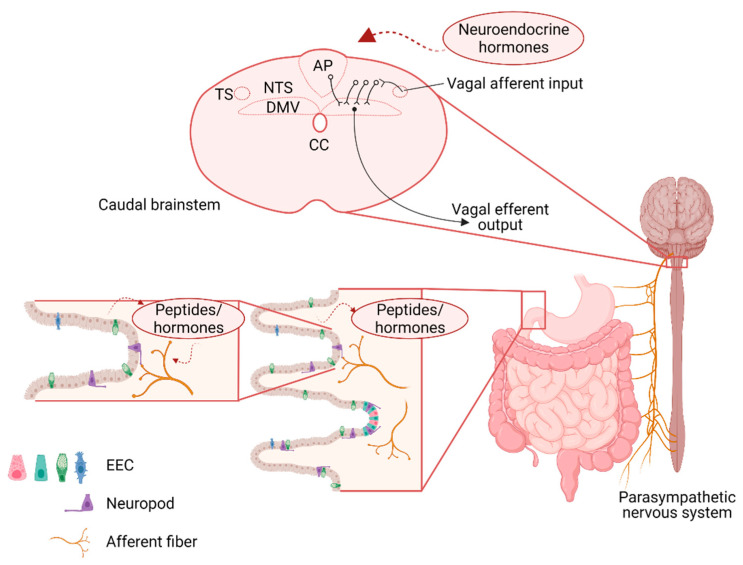
Schematic representation of the neuroanatomical connections between the gastrointestinal (GI) tract and brainstem vagal nuclei involved in the regulation of food intake. Sensory information from the GI tract is relayed centrally via the afferent vagus which responds both directly (via neuropods) and indirectly (via paracrine signaling) to gastrointestinal stimulation. At the level of the dorsal hindbrain, afferent vagal inputs enter the brainstem via the tractus solitarius (TS) and terminate on neurons of the nucleus of the tractus solitarius (NTS). The integrated signal is relayed from the NTS to the adjacent dorsal motor nucleus of the vagus (DMV) via catecholaminergic, glutamatergic, and predominantly gamma aminobutyric acid (GABA)-ergic, synapses. The DMV contains preganglionic parasympathetic motoneurons that relayed the resulting output signal to the GI tract via the efferent motor vagus. EEC—enteroendocrine cells; CC—central canal.

GI sensory information is relayed centrally via both vagal and spinal afferents. Spinal afferents, particularly those within the splanchnic nerves, relay low- or high-threshold mechanical and chemical information centrally and are considered primarily to be involved in nociceptive processing [1,2]. In contrast, the majority of interoceptive information is relayed centrally via vagal afferents, the cell bodies of which reside in the nodose and jugular ganglion. The central terminals of most of the gastrointestinal vagal afferents terminate within the brainstem at the level of the nucleus tractus solitarus (NTS), with a minority of terminals found in the area postrema (AP), the dorsal motor nucleus of the vagus (DMV), and the trigeminal islands [3]. Vagal afferent signaling is critical for the regulation of food intake and selective surgical denervation of GI vagal afferents has been shown to increase meal size and blunt the response to peripheral administration of GI peptides implicating vagal afferent signaling in the regulation of food intake [4] (Figure 1).

Gastric vagal afferents can be distinguished on the basis of their preferred response modality; mechanosensitive afferents, for example, innervate the GI smooth muscle and are activated by distention and stretch in response to food ingestion (reviewed in [5]). Recent studies have also shown that these tension-sensitive afferents can be further distinguished based upon their neurochemical phenotype, with glucagon-like peptide 1 receptor (GLP-1R) positive afferents innervating the stomach and oxytocin (OXT) positive afferents innervating the intestine [6]. Their distinct neurochemical phenotypes allow for selective experimental manipulation; optogenetic stimulation of these mechanosensitive afferents, for example, mimics central responses to gastric stretch, including the activation of distinct NTS populations and reduced food intake.

Mucosal afferents, in contrast, innervate the mucosal layer but do not make direct contact with the GI lumen. They are responsive to mucosal stroking and chemosensation and may play a role in the detection of food, particularly the size of food particles, within the GI lumen. The mucosal afferents are unresponsive to mechanical distention but are activated by intestinal nutrient infusion, and project to the NTS commisuralis [7]. Mucosal afferents also express distinct neurochemical phenotypes demonstrated by *Gpr65*-expressing neurons that display extensive innervation of the duodenal mucosa and are unresponsive to mechanical distention. Mucosal afferents are in close contact with enteroendocrine cells (EEC), and are respond to released gut endocrine peptides to induce an appropriate response in the vagal afferent neurons, and, subsequently, within the NTS, DMV, and the arcuate nucleus of the hypothalamus, [8] to regulate gastric emptying, pancreatic exocrine signaling, and intestinal fluid secretion, ultimately regulating food and caloric intake [5,9]. The presence of fat and protein within the intestinal lumen, for example, induces the release of cholecystokinin (CCK) from intestinal I-cells which leads to a pronounced vagal afferent-dependent reduction in meal size and is associated with activation of central activity in the hindbrain and hypothalamus [10,11,12]. The extensive connectivity of the NTS also provides pathways by which higher CNS centers can be activated in response to gut inputs; post-ingestive sucrose, for example, increases in activity in the left nodose ganglion and activates dopaminergic neurons in the ventral tegmental area (VTA) as a mechanism to reinforce food seeking behavior [13].

It should also be noted that recent studies have shown an additional specialized mechanism through which these EEC activate vagal afferents directly [14]. “Neuropods”, composed of long, axon-like projections from the basolateral side of the EEC, make direct synaptic connections with vagal afferent terminals (as well as other cells in the GI tract including enteric glia). Sucrose infusion in the distal intestine, for example, induces EEC glutamate release that activates vagal afferents in a synaptic transmission-dependent manner [14]. Subsequent studies have also shown that feeding-dependent neuropod activation induces activation of vagal afferents in the right nodose ganglion and a gut-induced ascending reward pathway via the parabrachial nucleus (PBN) to the Substantia nigra (SN) [13] (Figure 1).

### 1.2. Nucleus of the Tractus Solitarius (NTS)

The NTS is the major integrative center for various thoracic and abdominal sensory information and contains moderate viscerotropic organization of vagal afferent terminals (reviewed in [1]). Sensory inputs related to taste from the tongue, for example, project to the ventral NTS whereas afferents from the GI tract project to the caudal NTS [15,16]. Subnuclei within the caudal NTS form distinct neural populations relative to afferent inputs from the GI tract; gastric afferents innervate the NTS gelatinosus and commisuralis, while esophageal afferents innervate the NTS centralis [17,18]. Dendritic projections from these afferents have been shown to run the entire rostral-caudal extent of the NTS; however, providing a potential mechanism by which sensory inputs from the GI tract may be integrated with those from other viscera including gustatory areas as well as cardiothoracic and respiratory inputs [19].

Despite displaying immunoreactivity to a variety of neurotransmitters/modulators/hormones, GI sensory information is relayed principally, if not exclusively, via glutamate release from vagal afferent terminals and the activation of ionotropic glutamate receptors on NTS neurons [20,21]. Interestingly, while many studies have demonstrated that AMPA (and kainite) receptors are activated by vagal afferent-released glutamate, NMDA receptors may also be important in the regulation of food intake. Typically inactive at resting membrane potential due to the prominent Mg^2+^ ion block of the ligand-gated ion channel, NMDA receptors are not normally activated by synaptic release of glutamate [22,23]. Fourth ventricular microinjection of NMDA receptor antagonists, however, increase food intake following overnight fasting and abolish the satiating effects of peripheral CCK administration implying a role for these receptors in physiological feeding patterns [24,25]. Of note, electrophysiological studies investigating afferent-NTS glutamate transmission have shown that NMDA receptors are recruited following even relatively modest afferent stimulation frequencies (>5 Hz) and appear important in maintaining synaptic throughput when AMPA/kainate receptors are desensitized [26]. This implies that vagal activation in response to feeding is sufficient to induce NMDA receptor activation and likely either gates how much information from the viscera is projected to efferent pathways via the NTS or helps maintain reliable transmission across a wide range of stimulation frequencies. Afferent-released glutamate also activates metabotropic glutamate receptors (mGluRs) which have been identified at both pre- and post-synaptic locations, and heterosynaptic crosstalk has also been shown to modulate GABA release from NTS terminals [27,28].

Several studies have shown that spontaneous glutamate release from vagal afferents is modulated in an ongoing and tonic manner by a variety of neurotransmitters/modulators, including several neuropeptides contained within afferent terminals themselves [29]. Glucose, for example, regulates afferent glutamate release in a linear manner over a wide range of extracellular glucose levels via modulation of 5-HT_3_ receptor density [30,31,32,33,34]. The brainstem receives a dense serotonergic innervation from dorsal raphe nuclei, which tonically activates presynaptic 5-HT_3_ receptors; elevated glucose induces the trafficking of internalized 5-HT_3_ receptors to the membrane surface of vagal afferent neurons and their central terminals, increasing glutamate release; decreased glucose levels, in contrast, induces 5-HT_3_ receptor internalization, decreasing glutamate release [29,33,34,35]. Feeding neuropeptides, including cholecystokinin (CCK), ghrelin, cocaine- and amphetamine-regulated transcript (CART), and melanocortin concentrating hormone (MCH), amongst others, have all been shown to modulate afferent activity, hence regulate NTS neuronal excitation. Leptin, primarily produced by adipocytes [36], but also by gastric epithelial cells [37], has been shown to enhance glutamatergic transmission at the afferent-NTS synapse to reduce food intake [38], while activation of leptin-receptor expression NTS neurons has similar effects [39]. It is important to note that the dorsal vagal complex (DVC, i.e., the NTS, DMV and area postrema (AP)) is essentially a circumventricular organ with a leaky blood brain barrier and fenestrated capillaries [40]. Circulating neuropeptides, are significantly more likely, therefore, to activate central vagal neurocircuits [41,42]. Peripheral CCK, for example, has been shown to activate NTS neurons directly following a vagotomy [43]. “Feeding” neuropeptides released from enteroendocrine cells (EECs) may modulate vagal afferent activity at several discrete peripheral and central sites, therefore, in a prolonged temporal manner [29].

Neurons in the NTS can be identified by their distinct neurochemical phenotypes, principally glutamate, GABA, and norepinephrine (NE), but it should also be noted that many of the neuropeptides that activate vagal afferent terminals, including GLP-1 and CCK, are also expressed by neurons of the NTS and have been linked to the regulation of intake. For example, activation of GLP-1-containing NTS neurons reduces food intake [44,45] and modulates several autonomic responses. Furthermore, CCK- and dopamine beta hydroxylase (DBH)-containing NTS neurons form distinct neuronal populations that are activated by food intake in a vagal afferent-dependent manner, and induce downstream activation of calcitonin gene-related peptide (CGRP)-positive neurons in the PBN [46] to reduce food intake, while NTS neurons that co-express neuropeptide Y (NPY) and epinephrine (Epi) are distinct from those that express NE, and have opposing effects on vagal-afferent-dependent feeding behavior in a fasted state [47]. Taken together, it is clear that functionally-specific populations of NTS neurons play distinct roles in feeding behavior, and interrogation of these pathway-specific neurocircuits may increase our understanding of how such peripheral signals are integrated centrally to regulate energy homeostasis. Because of its projections to, and reciprocal innervation of, many other CNS areas involved in regulation of GI function and food intake the NTS plays a pivotal role in the central integration of gut inputs as well as efferent outputs to control, regulate, and modulate feeding behavior (Figure 1).

### 1.3. Dorsal Motor Nucleus of the Vagus (DMV)

Located ventral to the NTS, the dorsal motor nucleus of the vagus (DMV) contains the preganglionic parasympathetic motoneurons that provide efferent innervation to the esophagus, stomach, small intestine, and proximal colon. The subdiaphragmatic branches of the vagus nerve (5 branches in rodents: anterior gastric, posterior gastric, hepatic, celiac, and accessory celiac) have a medial-lateral columnar organization within the DMV [16,48,49]. While all DMV neurons are cholinergic, they differ in soma size and extent of dendritic arborization as per their efferent target organ [50,51,52]. DMV neurons innervating the stomach for example, are smaller and more excitable, hence more responsive to synaptic inputs and neuromodulation, than neurons that innervate the intestine. Vagal efferent DMV neurons that innervate the GI tract synapse onto postganglionic neurons that form one of two pathways; either excitatory cholinergic neurons that mediate smooth muscle contraction via muscarinic receptors or inhibitory neurons that mediate smooth muscle relaxation via nitric oxide (NO) or vasoactive intestinal peptide (VIP) (reviewed in [1,53]). Those involved in the excitatory pathway appear to originate more commonly in the medial and rostral DMV, with those involved in the inhibitory pathway more commonly found in the caudal DMV [54,55,56,57]. Through these opposing neural circuits, the DMV is able to exert fine-tuned control over GI functions, including gastric volume, contractile state, and rate of emptying, transit, and absorption, all of which influence feeding behavior.

While intrinsic biophysical properties of DMV neurons renders them spontaneously active pacemakers, firing at approximately 1 Hz [22], their activity is influenced by glutamatergic, catecholaminergic, and predominately tonic GABAergic inputs from the NTS [22,58,59]. Notably, however, the ability of this critical GABAergic synapse to be modulated—its “state of activation”—is controlled by presynaptic group II mGluR, activated by glutamate released from monosynaptic vagal afferent inputs. Tonic activation of these presynaptic receptors decreases cAMP levels, and prevents signaling through neurotransmitter receptors negatively coupled to adenylate cyclase (including μ-opioid, α2 adrenergic, NPY/PYY Y2, 5-HT1A receptors) [1]. Neuromodulators that activate cAMP-PKA signaling, e.g., CCK, GLP-1, corticotropin releasing factor (CRF), and insulin, increase the “state of activation” of this synapse, allowing GABAergic signaling to be inhibited, and increased activity of vagal efferent motoneurons [1]. Interestingly, in pathological states such as obesity where vagal afferent excitability is reduced, activation of the presynaptic mGluR is decreased and GABAergic signaling is enhanced rendering DMV neurons and vagal efferent output less excitable [60]. Leptin has also been shown to have direct inhibitory actions on DMV neurons, providing an additional mechanism in which this critical feeding peptide can alter GI functions, and food intake, via actions at central vagal sites [61].

Short-term neuroplasticity within NTS–DMV synapses has been identified recently as an important means by which food intake and caloric balance is regulated. Rodents respond to a calorically dense, palatable diet with an immediate short (24 h) period of hyperphagia before food intake is reduced after 3–5 days to restore caloric balance. This period of homeostatic regulation is associated with increased activation of synaptic NMDA receptors at the NTS–DMV synapse, leading to a subsequent increase in DMV neuronal excitability that could be driving this change in feeding behavior [23]. While studies have shown that gut peptides, including gastric inhibitor peptide (GIP) can induce central inflammation in pathological nutrient states [62], the increased activation of synaptic NMDA receptors coincides with an increase in neuroinflammatory markers in the brainstem, suggesting astroglia activation, which has been observed in the hypothalamus and nodose ganglion at this time point [62,63]. The full role these astroglia play in these central feeding centers, and how they can affect feeding behavior, in pathological conditions like obesity are the subject of renewed attention and focus, given their profound ability to alter synaptic efficacy and efficiency (Figure 1).

### 1.4. Area Postrema

The area postrema (AP) is a circumventricular organ situated in the fourth ventricle on the dorsal surface of the medulla, located adjacent to the NTS. As a circumventricular organ, it is highly vascularized with fenestrated capillaries and has dendritic projections that extend to the basal lamina side of the vascular endothelial cells, allowing it to be more exposed to circulating factors than the other regions of the brainstem that are involved in controlling feeding behavior [64]. Known classically as an emetic chemoreceptor trigger zone, AP neurons display receptors for many neuromodulators and immune mediators [65] including CCK, GLP-1, ghrelin, and PYY [64]. Administration of orexigenic gut peptides like CCK, GLP-1 and PYY alter AP neuron activation and excitability with subsequent effects on food intake [66,67,68,69,70] and ghrelin-dependent stimulation of feeding requires an intact AP [71].

**Figure 2:**
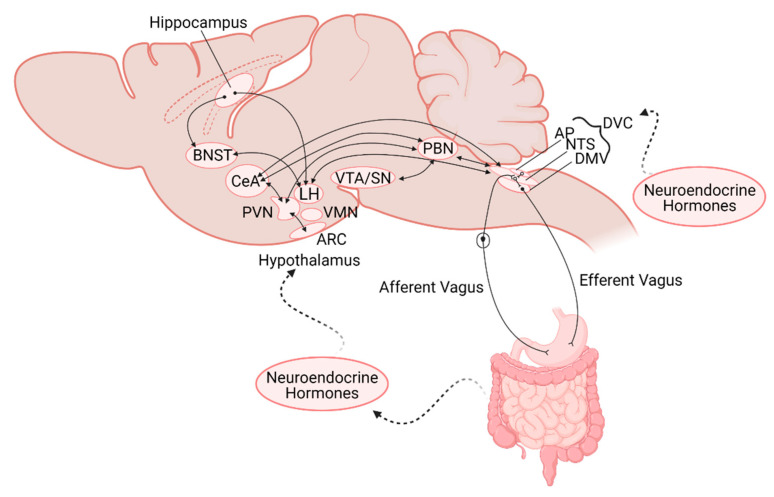
Schematic representation of the neuroanatomical connections between the gastrointestinal (GI) tract and central nervous system nuclei involved in regulation of GI functions and feeding. Note the location of the nuclei are not intended to be anatomically accurate. AP—area postrema; NTS—nucleus of the tractus solitarius, DMV—dorsal motor nucleus of the vagus; DVC—dorsal vagal complex; PBN—parabrachial nucleus; VTA/SN—ventral tegmental area/Substantia Nigra; PVN—paraventricular nucleus of the hypothalamus; VMH—ventromedial hypothalamus; LH—lateral hypothalamus; ARC—arcuate nucleus; CeA—central nucleus of the amygdala; BNST—bed nucleus of the stria terminalis.

In addition to its increased exposure to circulating factors, the AP also projects to, and receives input from, many regions involved in the regulation of nutrient status and feeding behavior. Basal dendrites of AP neurons receive direct inputs from gastric vagal afferents as well as having dense reciprocal connections with the NTS, PBN. AP neurons additionally receive inputs from the parvocellular neurons of the paraventricular and dorsomedial hypothalamus (PVN and DMH, respectively) [49,72], and have minor projections to the nucleus ambiguus, DMV, and cerebellum [49,72,73,74,75]. Due to its anatomical location, as well as its neurocircuitry, the AP serves a unique role as major integrative center for peripheral inputs, including from the GI tract, neuromodulatory circulating factors, and central areas involved in feeding control (Figure 1 and Figure 2).

### 1.5. Parabrachial Nucleus

Located in the dorsolateral pons, the PBN is responsible for relaying sensory inputs from the tongue and other viscera inputs from the NTS to forebrain structures involved in autonomic regulation [76]. Mechanical distension of the GI tract activates an NTS-PBN pathway that suppresses food and water intake [77]. In particular, the lateral PBN receives innervation from NE-, GLP-1-, and CCK-containing NTS neurons, and chemogenetic activation of these neurons induces satiation. Meal ingestion activates CCK- and GLP-1-expressing NTS neurons to excite CGRP-positive neurons in the lateral PBN [46,78]. In turn, these CGRP-positive PBN neurons project to, and activate, PKC-δ-positive neurons in the central amygdala (CeA) decreasing appetite [79]. Conversely, silencing of these same lateral PBN neurons delayed meal termination, subsequently increased meal size [80].

Beyond the homeostatic regulation of food intake as a response to the nutrient status of the organism, however, the lateral PBN has also been shown to be involved in a gut-mediated reward pathway. Activation of upper GI tract sensory vagal afferents induce dopamine release in striatal-related reward pathways, and the lateral PBN has been confirmed as the relay link between the DVC and the SN (Figure 2) [81]. Interestingly, it was CGRP- lateral PBN neurons that were activated by this vagal-dependent pathway, implying distinct subpopulations of lateral PBN neurons are responsible for mediating the rewarding response versus the meal termination response to food ingestion [81].

### 1.6. Cerebellum

The cerebellum, located posterior to and heavily connected with the brainstem, is canonically thought to be involved in motor control. Several lines of evidence, however, show that cerebellum also plays a critical role in the regulation of feeding behavior. Cerebellar lesions have been shown to alter feeding behavior and dysregulate blood glucose and body weight [82]. Although this may be caused by physical and motor impairment, it likely involves the well-established bidirectional links between the fastigial and interposed cerebellar nuclei with various hypothalamic nuclei including the lateral, ventromedial, dorsomedial and paraventricular hypothalamus (LH, VMH, DMH, and PVN, respectively) [83,84,85,86]. Interestingly, multiple studies have shown that projections from the cerebellum and the NTS converge on the same hypothalamic neurons, providing a means by which cerebellar signals can be integrated with gut inputs [82,87,88,89]. These same neurons have also been shown to respond to CCK, glucose, and ghrelin [87]. The cerebellum also innervates the DVC hence is able to modulate output responses to the GI [90,91].

### 1.7. Hypothalamus

The hypothalamus lies rostral to the brainstem, making up the floor of the third ventricle. Composed of many subnuclei, the hypothalamus has pivotal roles in the autonomic control of thirst, temperature, biological rhythms as well as feeding and glycemic regulation. Focusing on its role in the integration of sensory gut inputs and the regulation of feeding patterns, the hypothalamus receives ascending gut-related projections from the NTS as well as responding directly to circulating feeding-related factors.

Located in the basal hypothalamus adjacent to the arcuate (ARC) nucleus, the median eminence is a circumventricular organ [41,42] that has been shown to be responsive to circulating gut peptides, including ghrelin, which activates orexigenic ARC neurons (expressing Neuropeptide Y (NPY) and agouti-related peptide (AgRP) and inhibits anorexigenic neurons (pro-opiomelanocortin (POMC)- and melanocyte-stimulating hormone (MSH-containing) in order to stimulate feeding behavior [92]. Peripheral administration of Peptide YY (PYY), a gut peptide produce by L-cells in response to luminal contents, reduces food intake, which is at least partially driven by PYY acting on its receptors in the ARC to inhibit NPY/AgRP neurons [93,94]. Thus, circulating gut peptides may modulate feeding behavior by regulating the balance of NPY/AgRP orexigenic and POMC/MSH anorexigenic activation.

Beyond exposure to circulating factors, the ARC receives input from other hypothalamic nuclei as well as the NTS, the locus ceruleus, reticular formation, and bed nucleus of the stria terminalis (BNST; Figure 2 [95,96]. Noradrenergic projections from the NTS have been shown to mediate feeding behavior through direct projections to AgRP neurons in the ARC to stimulate feeding in a hypoglycemic state [97]. Conversely, TH/Prolactin releasing peptide (PrPR)-containing NTS neurons inhibit ARC AgRP neurons in response to high protein meals [98]. In addition to its projections to other hypothalamic and brainstem nuclei, the ARC also sends descending projections to the DVC, which allows for the direct regulation of visceral efferent outputs [95]. This connection with the DVC appears to play an important role in leptin’s ability to modulate food intake, as loss of leptin receptor signaling at the DVC reduced the ability of leptin to decrease food intake through its actions at the hypothalamus [99].

The PVN has also been implicated in affecting feeding behavior via gut-dependent inputs. The PVN has reciprocal connections to may central feeding-related nuclei including the ARC, NTS, PBN, BNST, and CeA (reviewed in [100]). Peripheral CCK administration, for example, excites MC4R-containing PVN neurons, subsequent to activation of TH-positive NTS neurons, resulting in rapid satiation [101]. The PVN additionally sends descending projections to the DVC and PBN which have profound effects on GI outcomes, including feeding patterns [100,102,103]. PVN-derived CRF, for example, activate DVC neurons to modulate gastric motility and emptying [104,105], while oxytocin (OXT) released decrease gastric motility through a vagal-dependent pathway [106,107,108].

A subpopulation of the LH, composed of orexin/hypocretin neurons, has been shown to form synaptic connections with NTS neurons and modulate food intake [109]. This provides an avenue for hypothalamic orexin neurons, classically thought of as mediating attention and arousal, to affect the brain-gut axis [110]. The suprachiasmatic nucleus (SCN), the master clock of the brain, is known to control the daily rhythms of many biological functions. Ablation of the SCN leads to a loss of the feeding-fasting cycle, with no changes in food intake or meal frequency, implicating it in control of meal timing [111]. SCN neurons are also responsive to glucose and ghrelin, implying modulation by nutrients and gut-related signal [112] but it should be noted that the SCN does not appear to become entrained to changes in diet, implying there are other regions (including vagal afferents and the NTS) that are likely to play a larger role in the control of food rhythms [111].

### 1.8. Hippocampus

Beyond autonomic and homeostatic control, feeding is also regulated by higher cognitive centers. The memory of recent meals can control initiation, frequency, and size of future meals, as well as encoding information regarding where food was available. The hippocampus, split anatomically into dorsal and ventral regions, is known to be involved in these memory formations, placing it as a potentially critical central region for controlling food intake. Indeed, hippocampal neurons are activated by gastric distention [113] in addition displaying receptors for many GI peptides including CCK, GLP-1, ghrelin, and other endocrine factors like insulin, leptin, and amylin [114]. Although monosynaptic inputs from the NTS have not been identified, the hippocampus receives inputs from other brainstem regions including the locus ceruleus and dorsal raphe nuclei that may relay information from the NTS (Figure 2) [114]. The hippocampus has descending projections to other central regions known to be involved in food intake, including the BNST and LH [115,116,117]. Inactivation of ventral hippocampal neurons decreases inter-meal intervals, increase meal size, frequency, and total intake [116,118,119,120,121,122]. In contrast, the dorsal hippocampus is necessary for the episodic memory of a recently eaten meal, influencing the inter-meal interval and amount eaten at that next meal [123,124].

### 1.9. Amygdala

The amygdala, located in the temporal lobe, lies adjacent to the inferior horn of the lateral ventricle and medial to the hypothalamus. The amygdala can be broken down into substructures that include the central amygdala (CeA) and the extended amygdala that includes the bed nucleus of the stria terminalis (BNST). The CeA receives projections from the PBN and the PVN and sends descending projections to the hypothalamus and brainstem in order to mediate physiological responses to fear and anxiety (Figure 2) [125]. The CeA has also been suggested to play a more direct role in the regulation of feeding behavior, however. A subpopulation of neurons (PKC-δ-positive) in the lateral CeA are activated by anorexigenic signals from the gut such as CCK, and play a critical role in mediating the subsequent inhibition of food intake [79]. A second population of neurons in the CeA (PKC-δ-negative/5-HTR-positive) have also been shown to promote feeding behavior through inhibitory projections to the PBN. The BNST-LH neurocircuit has also been implicated in controlling feeding behavior [126]. Thus, while inter-amygdala circuits and descending control from the amygdala have shown to mediate feeding behaviors, the exact mechanisms and physiological relevance are still subject to investigation.

## 2. Pathophysiology

### 2.1. Obesity, Diabetes, Inflammation

Several studies have highlighted changes in vagal afferent responsiveness and signaling following high fat diet (HFD) exposure and diet-induced obesity (DIO), including a decreased vagal afferent response to mechanical stretch of the stomach and neuroendocrine peptides including CKK, GLP-1, leptin, and serotonin, as well as impaired responses to glucose [31,127,128]. HFD exposure is associated with altered gut peptide secretion, which mimics a perpetual fasting phenotype at the level of the vagal afferents, exacerbating the changes in GI function and food intake seen in the development of obesity [129]. HFD exposure also modulates central neurocircuits, both at the level of the brainstem and hypothalamus. Within vagal neurocircuits, the decrease in vagal afferent sensitivity responsiveness would be reasonably expected to decrease afferent glutamate release centrally. Indeed, HFD is associated with decreased afferent-dependent activation of presynaptic metabotropic glutamate receptors (mGluR) on inhibitory GABAergic NTS–DMV synapses, increasing the inhibitory drive to vagal efferent motoneurons. DMV neurons are also less excitable and less responsive to satiety neuropeptides such as CCK and GLP-1 following DIO [60,130,131]. In fact, these effects can be observed even before the onset of weight gain, suggesting that the HFD itself is responsible for at least some of these neuronal and synaptic changes, independent of the increased adiposity associated with obesity [23,132].

Another interesting phenomenon, well documented by Levin and colleagues, has shown that HFD-fed male Sprague-Dawley rats separate into two populations, one that become hyperphagic and obese, and another that maintain their eating patterns and resist weight gain [133]. Of note, hindbrain catecholaminergic ventrolateral medulla and NTS neurons that relay GI-sensitive inputs to the PVN, DMH, and ARC within the hypothalamus are disrupted in the obesity-prone population, highlighting neurodevelopmental differences that may underlie the propensity to develop DIO [134].

### 2.2. Developmental Modulation of Central Neurocircuits

Central feeding circuits develop during the embryonic period, but are subject to significant development maturation in the early postnatal period. In rodents, descending autonomic inputs from the hypothalamus, CeA, BNST, and cortex reach the DVC in the first postnatal week, marking this as a critical window during which early life experiences can shape the connectivity of these central feeding nuclei [135]. Several studies have demonstrated that maternal and early postnatal diet affects the development of central feeding-related neurocircuits [136,137]. Offspring exposed to perinatal HFD have increased body weight and an increased risk for developing obesity in adulthood [138]. At the NTS–DMV brainstem synapse, for example, there is a attenuation of the developmental switch in GABA_A_ receptors subunit composition that alters the channel kinetics and subsequently increases inhibitory drive from the NTS that is associated with decreased gastric motility [139], although the long-term effects of this developmental delay on feeding behavior needs to be determined. Hypothalamic-DVC neurocircuits are also altered by maternal HFD exposure [140]. Elevated levels of orexigenic neuropeptides are present after maternal HFD that resembles the phenotype observed following adult HFD exposure [138], possibly due to transcriptional changes at the neuronal level [141,142]. Postnatal HFD exposure has also been shown decrease, while postnatal undernutrition increases, AgRP ARC fiber density [143,144]. Taken together, postnatal diet has the ability to influence the responsivity and developmental maturation of hypothalamic neurons, including their descending projections to the DVC, which may have long term and persistent consequences on central responses to gut inputs and dysregulated feeding behavior.

### 2.3. Neurological Disorders

Because of the extensive interconnectivity of brainstem, midbrain, and higher CNS centers with the DVC and brainstem neurocircuitry involved in the regulation of GI functions, several neurological, developmental, and behavioral disorders are associated with alterations in gut functions, including those involved in the regulation of food intake and energy balance.

Stress has been defined as a stimulus or event that challenges the physiological and psychological homeostatic state of an individual [145,146,147]. Critical to the central stress response is activation of the hypothalamic-pituitary-adrenal (HPA) axis, including activation of descending hypothalamic-DVC projections which modulate GI functions. In fact, functional GI disorders correlate with stress, including function dyspepsia and irritable bowel syndrome, and stress is known to exacerbate GI dysfunction in vulnerable individuals. [148,149]. Of note, while CRF- containing PVN-DVC neurons play a pivotal role in the effects of stress on visceral organs, CRF antagonists do not appear to modulate GI functions in non-stressed individuals. The effects of acute stress to delay gastric emptying and accelerate colonic transit can be mimicked by a temporally restricted exposure to CRF. Adaptation to prolonged stress, however, requires a more profound adaptive neuroplasticity, and several studies have shown that this involves upregulation of descending PVN-DVC OXT inputs, which play a major role in the recovery of GI functions [147,150].

Autism spectrum disorder (ASD) is a neurodevelopmental disorder characterized by behavioral disturbances such as repetitive behaviors and impaired social behaviors. Beyond changes to social behavior, these patients also exhibit altered eating patterns where children with ASD are considered very selective eaters (picky eaters), with aversions to particular food colors, smells, and textures, amongst other characteristics [151,152]. An overall change in total caloric intake is not reported, however, implying that satiety mechanisms may not necessarily be dysfunctional [153]. GI dysfunctions are reported in up to 91% of individuals with ASD, including bloating, constipation, diarrhea, and gastroesophageal reflux [154], indeed, this picky eating has been hypothesized to be an adaptive behavior to avoid GI discomfort [153,155]. Although ASD has been associated with many changes in the gut microbiome, central cranial nerve abnormalities, including motor nuclei in the brainstem and reduced vagal tone, have also been reported [156,157]. Peripheral OXT levels are also disrupted which, as discussed previously, may also contribute to the GI dysregulation and alterations in food intake, in addition to the well-recognized role in impaired social behaviors [158,159].

Parkinson’s disease (PD) is a neurodegenerative disorder characterized by severe degeneration of dopaminergic neurons in the substantia nigra pars compacta (SNpc). It is considered a movement disorder marked by bradykinesia, rigidity, tremor, and gait/posture disorders. However, PD patients also experience a wide array of prodromal symptoms that include sleep disorders, orthostatic hypotension, and GI dysfunction [160,161,162]. Disorders of the GI tract include dysphagia, nausea, delayed gastric emptying, dysmotility, constipation, and fecal incontinence that can be detected in some patients many years before the onset of motor symptoms [163,164]. Interestingly, α-synuclein protein aggregates, known as Lewy bodies, have been shown to be present in the myenteric neurons within the ENS as well as the vagal motor neurons in the DMV, providing a potential explanation of the manifestation of prodromal GI dysfunction [165]. The recent discovery of a monosynaptic nigro-vagal pathway provides a pathway by which dopaminergic projections from the SNpc modulate DMV neuronal activity, hence GI functions [166,167], as well as providing an anatomical means by which an ENS-DVC-SNpc neurocircuit may underlie the pathophysiology of PD [168].

## 3. Conclusions and Future Directions

The regulation of food intake is a result of a coordinated and intricate series of short- and long-term responses to peripheral and central nervous system signals. Although circulating factors can modulate the activity of circumventricular nuclei directly, sensory inputs from the gut are relayed centrally via the afferent vagus nerve, terminating in a pathway-specific manner predominately within the NTS. Because of its reciprocal connections with other brainstem, midbrain, and forebrain nuclei, the NTS plays a major role in consolidating responses from across the CNS and relaying an integrated signal to the adjacent DMV to control the vagal motor output to the GI tract. This interconnectivity has led to the increased appreciation that many disorders of the CNS are accompanied by dysregulated central control of GI dysfunction, including disruptions in feeding, metabolism, and energy balance which significantly affects many patient’s quality of life.

The advent of novel and emerging molecular and genetic techniques has allowed identification of discrete gut–brain neurocircuits involved in particular GI responses, and affirmed that many such neurocircuits are exquisitely specialized as per their physiological response. Using a multi-functional approach, the gut–brain axis has been shown to determine preference for sugar (but not artificial sweetener, hence not sweet-taste) via post-ingestive mechanisms involving afferent vagus nerve-mediated activation of the caudal NTS [169]. While the association of distinct and different neurocircuits for sweet taste in the tongue and gut–brain axis may reinforce the preference for sugar-rich foods, development of an artificial sweetener that can co-activate both pathways may well reduce the drive for sugar consumption [169]. Neuroplasticity is the hallmark of learning, memory, and homeostatic adaptation to ongoing physiological conditions and alterations within the gut–brain axis is no exception. As described above, decreased sensiivity and responsiveness in vagal afferents occurs following high fat diet exposure or the development of diet-induced obesity. Hypersensitivity within vagal afferents, however, may be responsible the unwelcome GI symptoms that result from ingestion of food allergens. The recent finding that bacterial infections cause the breakdown of oral tolerance to food antigens via a histamine H1 receptor-mediated sensitization of visceral afferents may provide a mechanistic basis by which food sensitivities and/or allergies can occur [170]. Furthermore, not only may this provide therapeutic targets by which inappropriate immune responses may be addressed, it may also uncover avenues by which visceral hypersensitivity disorders that are exacerbated by food ingestion (such as irritable bowel syndrome and functional dyspepsia) may be alleviated. Of note, the intestinal EEC are also equipped to function as “sentinels” within the intestinal environment and release cytokines in response to microbial metabolites and pathogens [171]; the direct synaptic connection between EEC and vagal afferent nerve endings suggests an additional role for neuropods in rapid signaling of mucosal immune responses via the gut–brain axis, or even in axon reflex responses to sensory stimulation [172].

Many challenges and barriers still exist to understanding the mechanisms by which central neurociruits regulate food intake in response to gut inputs. The central connectome of the gut–brain axis has yet to be fully elucidated, particularly in regard to pathway-specific neurocircuits. While studies from several groups have provided a great deal of information regarding vagal efferent control of GI functions in both physiological and pathophysiological conditions, there is surprisingly little phenotypic characterization of vagal efferent neurons involved in different visceral functions. Questions still remain regarding the validity of many animal models used to investigate complex human physiologial systems; while the use of transgenic animals has clearly pushed our understanding of nutrition, feeding and the role of the gut–brain axis forward, using such a reductionist approach to answer polygenic pathophysiological problems is not without translational difficulties. Little is known about how the gut–brain axis or the responsiveness of central neurocircuits to visceral inputs vary across the life span or during ageing and, despite strong clinical evidence that the perinatal envionment is critically important to offspring outcomes, few studies have investigated how early life events alter the development of feeding related neurocircuits. Nevertheless, increasing awareness of the crucial role that the CNS plays in modulating GI sensory and motor functions should encourage continued investigations into the reciprocal ‘big brain–little brain’ partnership.
